# Gamma-Glutamyltransferase Activity (GGT) Is a Long-Sought Biomarker of Redox Status in Blood Circulation: A Retrospective Clinical Study of 44 Types of Human Diseases

**DOI:** 10.1155/2022/8494076

**Published:** 2022-06-06

**Authors:** Cui Bai, Meng Zhang, Yiran Zhang, Yixiong He, Huaiqian Dou, Ziyue Wang, Zhiliang Wang, Zipu Li, Lijuan Zhang

**Affiliations:** ^1^Systems Biology & Medicine Center for Complex Diseases, Center for Clinical Research, Affiliated Hospital of Qingdao University, Qingdao 266003, China; ^2^Department of Pediatrics, Affiliated Hospital of Qingdao University, Qingdao 266003, China; ^3^Shandong Institute of Orthopedics and Traumatology, The Affiliated Hospital of Qingdao University, Qingdao 266003, China; ^4^Cheeloo College of Medicine, Shandong University, Jinan 250012, China; ^5^Department of Computer Science, Viterbi School of Engineering, University of Southern California, Los Angeles, CA 90089, USA; ^6^Heart Center, Qingdao Women and Children's Hospital, Qingdao 266034, China

## Abstract

**Methods:**

The clinical GGT data from 168,858 patients with 44 diseases and 132,357 healthy control in the clinical laboratory of our hospital over the past five years were retrieved. All data were analyzed with SPSS, RStudio V.1.3.1073, and python libraries 3.8.

**Results:**

Thirty-eight out of 44 diseases had significantly increased (*p* < 0.001) circulating GGT activities, whereas gastric cancer, anemia, renal cyst, cervical cancer, preeclampsia, and knee-joint degenerative diseases had significantly decreased (*p* < 0.001) GGT activities compared to the healthy control. ROC analyses showed that GGT was an excellent biomarker for liver cancer (AUC = 0.86), pancreatitis (AUC = 0.84), or hepatic encephalopathy (AUC = 0.80). All pancreas-related diseases had more than 8-fold increases in GGT activity span than the healthy control, while pancreatic cancer had a 12-fold increase (1021 U/L vs. 82 U/L). The knee-joint degenerative disease had the lowest median and narrowest GGT activity range (63 U/L). Furthermore, most diseases' lowest to highest GGT activities were beyond the healthy control in both directions.

**Conclusions:**

Thirty-eight out of 44 diseases were in overall oxidative states defined by the increased GGT median values. In contrast, knee-joint degenerative disease, gastric cancer, anemia, renal cyst, cervical cancer, and preeclampsia were in overall antioxidative states. Moreover, most diseases swing between oxidative and antioxidative states, evidenced by the increased lowest to highest GGT activity ranges than the healthy control. Liver- and pancreas-related abnormalities were responsible for significantly increased GGT activities. Our overall results suggested that circulating GGT was a redox status biomarker.

## 1. Introduction

The vast majority of organisms on Earth depend on oxygen to generate life energy. However, oxygen also produces harmful reactive oxygen species (ROS). Keeping redox equilibrium is essential for all life forms. In human physiology, homeostasis is the state of steady internal, physical, pH, and redox conditions maintained by all tissue/organ systems. It is generally believed that ROS is primarily responsible for oxidative stress that causes various diseases in the past. However, ROS has a dual role; whether it acts as a harmful, protective, or signaling factor depends on the balance of the redox state at the right time and in the right places [1–3]. Developing biomarkers that are indicative of redox status will be helpful for patient care in modern medicine.

Glutathione (GSH) is a significant antioxidant to serve as a scavenger of ROS inside and outside cells in the human body [4]. Gamma-glutamyltransferase (GGT) is the enzyme that cleaves gamma-glutamyl residue from GSH to produce cysteine-glycine. Cysteine is then generated when the cysteine-glycine dipeptide is further cleaved. Cysteine-glycine and cysteine are transported back to cells for GSH resynthesis to maintain the reducing environment inside of cells. Outside the cells, the GGT-generated products, cysteine-glycine, and cysteine reduce ferric iron Fe (III) to ferrous Fe (II) more efficiently than GSH. ROS, including superoxide radical and hydrogen peroxide, are Fe (II) coproducts upon Fe (III) reduction [5]. Overall, GGT serves as both an antioxidant and a prooxidant [6]. Since discovering its prooxidant property, GGT in blood circulation is proposed to modulate oxidation-reduction (redox) equilibrium within the cells and their surroundings [6]. Thus, circulating GGT activity might be an ideal candidate for redox biomarkers.

Inflammation follows virtually any tissue/organ damage *in vivo*. Most inflammatory cells carry ROS as a bioweapon to create an oxidative environment to kill pathogens. Since keeping redox equilibrium in check is critical for life, we hypothesized that the unique anti- and prooxidative properties might make GGT a general redox biomarker in blood circulation. Indeed, studies have shown that circulating GGT can be biomarkers for various human diseases [7].

Previous studies on GGT as a biomarker were mainly limited to one type of disease at one time. Circulating GGT in 44 different human diseases and the healthy control were systematically compared and analyzed as a redox biomarker in the current study. A total of 301,215 clinical lab test results of serum GGT activities from healthy individuals and patients with 44 different diseases were retrieved and analyzed during the past five years. We concluded that circulating GGT was a biomarker of the redox status of various illnesses.

## 2. Methods

### 2.1. Clinical Data Collection

After obtaining approval from the Hospital Ethics Review Board of Qingdao University (QYFYWZLL26214), we were allowed to retrieve the electronic medical records and lab data of serum GGT activities of both healthy individuals and patients with clinically defined diseases from the clinical laboratory of the Affiliated Hospital of Qingdao University during the past five years. All research was performed by relevant guidelines/regulations, and informed consent was obtained from all participants or their legal guardians. All diseases include patients at different stages of disease development with or without medical interventions. Since the testing machine, the operators, other uncontrollable factors during the serum sample handling process, or life-saving medical interventions, such as the blood sample which was collected right after surgery, could generate extraordinarily high or low GGT test values; we excluded the 2.5% lowest and 2.5% highest GGT test values from each disease to have 95% GGT data during the past five years for analysis. As a result, 168,858 GGT activities from patients suffering from 44 diseases and 132,357 GGT activities from the healthy control were used for data analysis.

### 2.2. Measurements of Serum GGT Activities

The clinical lab in our hospital used a GGT assay kit (lactic acid substrate method, Beijing Leadman Biochemistry Joint Stock Limited Company, Beijing, China) for serum GGT activity measurement according to the manufacturer's instructions, which was adapted to the automated biochemistry analyzer (Hitachi HCP­7600, Japan). GGT catalyzes a reversible conversion of gamma-glutamyl-paranitroaniline and diglyceride to paranitroaniline and gamma-glutamyl diglyceride. The GGT activities were directly proportional to the paranitroaniline. The GGT activity is measured on the absorbance change of the paranitroaniline at 405 nm.

### 2.3. Statistical Analysis

All data retrieved was analyzed with RStudio V.1.3.1073 (RStudio, Boston, USA), Review Manager V5.4 (Copenhagen: The Nordic Cochrane Centre, The Cochrane Collaboration), and python libraries 3.8 (Anaconda Software Distribution). The results were demonstrated as both the mean ± standard deviation (SD) and median. A standard *t*-test was used to compare the clinical characteristics of subjects in the specific disease and control groups. Since a small proportion of extremely large or small and the nonstandard Gaussian distribution of GGT values observed in most of the diseases, we also used 2.5 percentile (Q2.5), 25 percentile (Q25), median or 50 percentile (Q50), 75 percentile (Q75), and 97.5 percentile (Q97.5) for data presentation and analysis. Median levels of circulating GGT activities between groups were compared utilizing the Mann–Whitney *U*-test. Groups were compared using the Kruskal-Wallis test (a nonparametric one-way ANOVA). Logistic regression was used to test the interactive effects of other variables on the observed association. *P* < 0.05 was considered statistically significant.

### 2.4. ROC Analysis

SPSS v26 (IBM, Armonk, US) was used to plot receiver operating characteristic (ROC) curves to evaluate GGT as biomarkers for 44 different diseases. The same numbers of healthy individuals were randomly selected from the pool of 132,357 healthy individuals to match the sex and ages of patients suffering from each specific disease. The Youden index is defined for all points of a ROC curve, and the maximum value of the Youden index is used as a criterion for selecting the optimum cut-off point. We selected the maximum *J* value as the Youden index out of all *J* values. The maximum value of Youden's index for the ROC curve was used to calculate the sensitivity and specificity for each disease. The AUC values of ≤0.5, 0.5 to <0.7, 0.7 to <0.8, 0.8 to <0.9, and ≥0.9 indicate no, poor, acceptable, excellent, and outstanding biomarkers, respectively. The *P* ≤ 0.05 was considered statistically significant.

### 2.5. The Two-Component Analysis

We conducted the two-component analysis based on the published reports [8]. We quantified the statistical features of the circulating GGT levels in each of 44 diseases, including Q2.5, Q25, Q50, Q75, Q97.5, and mean ± SD. The obtained statistical features were further standardized by decoupling the median and scaling to unit variance [8]. Applying the singular value decomposition of the data to reduce the dimensionality and extract the principal component [9]. We projected the data to a lower-dimensional space while retaining the essence of the given statistics. The correlation between each disease on the statistical features is proportional to their vector distance.

## 3. Results

Based on the lab data of serum GGT activities retrieved from the clinical lab of our hospital over the past five years, we calculated and listed the number of cases, median (interquartile ranges), mean (standard deviation (SD)), and *P* value in comparison to the healthy control for each of the 44 diseases in Table 1.

The patients suffering from the renal cyst, cervical cancer, preeclampsia, and knee-joint degenerative diseases had lower mean values of serum GGT activities than the controls with statistical significance (*P* < 0.001, Table 1). The other 40 types of diseases had higher mean values of serum GGT activities than the healthy controls with statistical significance (*P* < 0.001, Table 1). Moreover, among the 44 diseases studied, patients with pancreatic cancer had the highest mean value of serum GGT activities (139.0 U/L).

The extreme GGT activity ranges from the lowest to the highest values were the characteristics of the liver- and pancreas-related diseases (Table 1). Pancreatic cancer had the broadest GGT activity ranges in its patient population (1,160 cases). The extreme GGT activity ranges were not observed in the diseases with the largest patient populations tested (Table 1), such as lung cancers (10,930 cases), coronary heart disease (22,117 cases), type 2 diabetes (11,629 cases), and the healthy control (132,357). Thus, the broadest GGT activity range was pancreatic cancer-specific.

**Figure 1:**
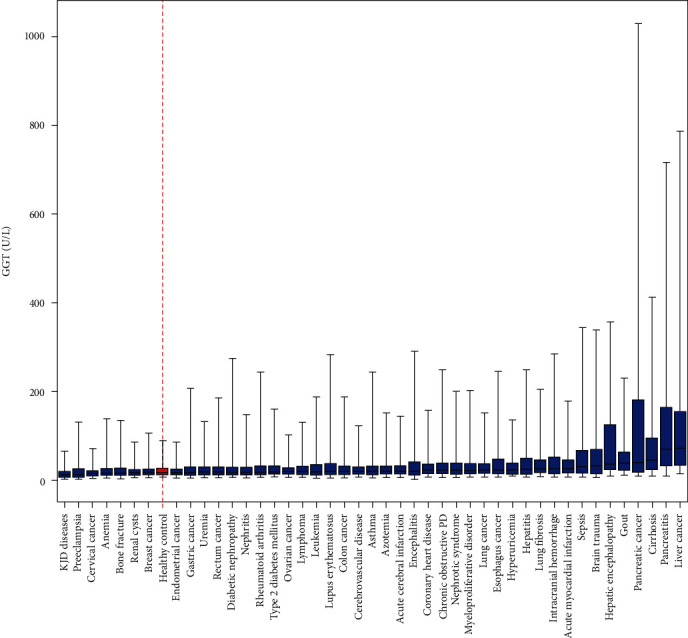
Serum GGT activities in 44 different types of diseases. The data were sorted in an ascending order according to the median values. Chronic obstructive PD: chronic obstructive pulmonary disease.

To better visualize the results, we made a box plot of serum GGT in 44 diseases with the minimum (Q2.5), the first quartile (Q25), the median (Q50), the third quartile (Q75), and the maximum (Q97.5) marked for 44 diseases and the healthy control (Figure 1). Since the testing machine, the operators or other uncontrollable factors during the serum sample handling process could generate extraordinarily high or low GGT test values, we excluded the 2.5% lowest and 2.5% highest GGT test values from the data analysis.

Based on the pattern shown in Figure 1, here were some of the observations. Among all diseases, knee-joint degenerative diseases (KJDD) had the narrowest GGT activity range (63 U/L, the maximum GGT activity value at Q97.5 minus the minimum GGT activity value at Q2.5%). Moreover, the minimum and the maximum GGT activities in knee-joint degenerative diseases were lowest compared to the healthy control and other diseases. The GGT activity span from Q2.5 to Q97.5 increased 12-fold in pancreatic cancer than the healthy control (1021 U/L vs. 82 U/L). Moreover, all pancreas-related diseases had more than 8-fold increases in GGT activity span compared to the healthy control.

**Figure 2:**
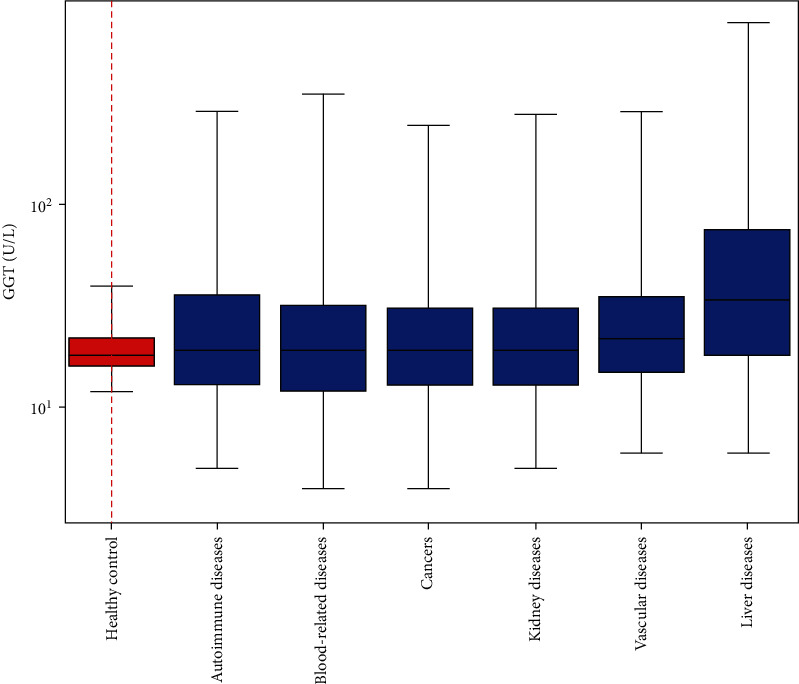
GGT activity distributions in six categories of diseases and the healthy control. Autoimmune diseases: lupus erythematosus, rheumatic arthritis; blood-related diseases: sepsis, leukemia, myeloproliferative disorder, lymphoma, multiple myeloma, and anemia; cancers: lung cancer, esophagus cancer, ovarian cancer, colon cancer, rectum cancer, endometrial cancer, breast cancer, gastric cancer, and cervical cancer; kidney diseases: nephrotic syndrome, azotemia, nephritis, uremia, diabetic nephropathy, and renal cyst; vascular diseases: acute myocardial infarction, intracranial hemorrhage, coronary heart disease, acute cerebral infarction, cerebrovascular disease; liver diseases: liver cancer, hepatic encephalopathy, cirrhosis, and hepatitis. Due to its unique GGT activity distribution, pancreatic cancer was not included in the category of cancers. Distinctive illnesses, including pancreatitis, brain trauma, encephalitis, lung fibrosis, chronic obstructive pulmonary disease, asthma, gout, hyperuricemia, type 2 diabetes mellitus, bone fracture, and knee-joint degenerative diseases, were classified into other diseases and did not included in this figure for comparison.

To comprehend the heterogeneity of GGT activities among different diseases, we divided the 44 diseases into six categories, including liver diseases, cancers, autoimmune diseases, vascular diseases, blood-related diseases, and kidney diseases with different color-code. GGT activity distributions in 6 categories of diseases and the healthy control are plotted in Figure 2. Among the six categories of diseases, liver-related diseases had the highest, whereas autoimmune diseases had the lowest Q50 values. Most importantly, the GTT activity ranges from either Q2.5 to Q97.5 or Q25 to Q75 (blue boxes) for all six category diseases significantly exceeded the healthy control in both directions, which indicated that GGT activities were over down- and upregulated in all diseases.

**Figure 3:**
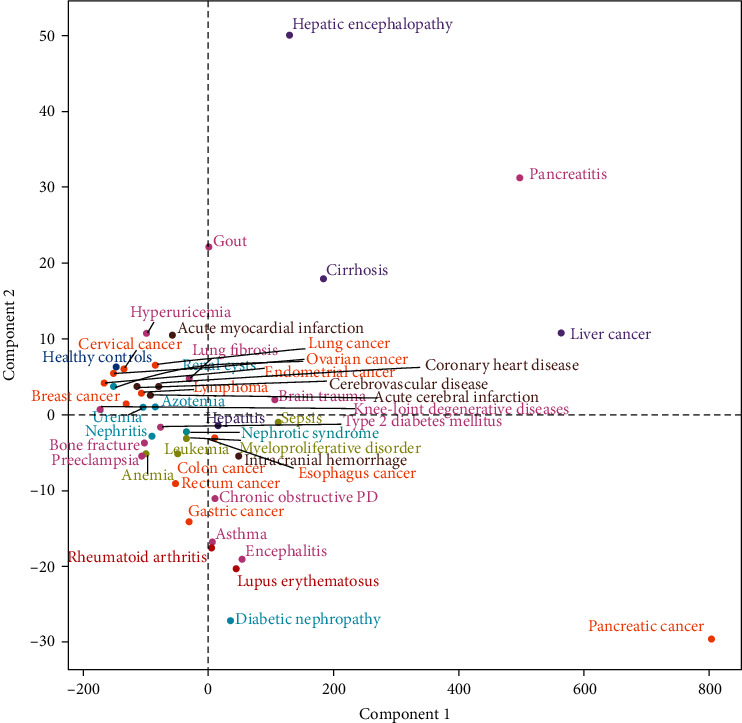
The changes in serum GGT activities had common features for the same class of diseases. The 44 diseases were divided into seven categories, including liver diseases (marked in purple), cancers (marked in orange), autoimmune diseases (marked in red), vascular diseases (marked in dark-red), blood-related diseases (marked in light-green), kidney diseases (marked in light-blue), and other diseases (marked in pink). The statistics features of the GGT activities for each of 44 diseases, including the mean, standard deviation, Q2.5, Q25, Q50, Q75, and Q97.5, were quantified. The obtained statistics features were further decoupled into two major components and presented.

We then quantified the statistics features of the GGT activities for each of 44 diseases, including the mean, standard deviation, Q2.5, Q25, Q50, Q75%, and Q97.5. The obtained statistical features of all diseases are shown in Figure 3.

The apparent clustering of the same category of diseases, such as kidney diseases including nephrotic syndrome, nephritis, azotemia, renal cyst (at the left side of the chart), autoimmune disease including rheumatic arthritis, lupus erythematosus (at the low right of the chart), blood-related diseases, including leukemia, myeloproliferative disorder, anemia (at the down left of chart), cardio-cerebrovascular diseases including acute myocardial infarction, coronary heart disease, acute cerebral infarction, cerebrovascular disease (at the upper left of chart), were observed based on the statistical analysis. Except for liver and pancreatic cancers, most solid cancers were clustered on the left side of the chart. Intracerebral hemorrhage was the only cardio-cerebrovascular disease not clustered at the upper left chart. Interestingly, all gynecologic cancers were clustered at the upper left side of the chart, even though cervical cancer had lower serum GGT activities.

**Figure 4:**
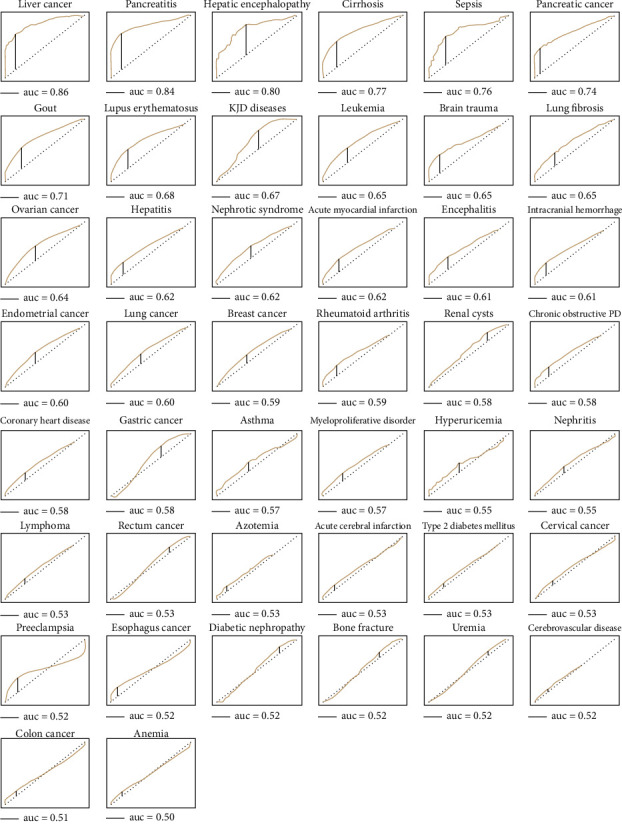
Serum GGT activity as a biomarker for 44 types of human diseases was assessed by receiver operating characteristic (ROC) curve analysis. The ROC curves were sorted in descending order according to the AUC values. Vertical line (J), maximum value of Youden's index for the ROC curve, was used to calculate sensitivity and specificity for each of the 44 diseases. We summarized the area under the curve (AUC), accuracy, sensitivity, and specificity for all diseases in Figure 5.

**Figure 5:**
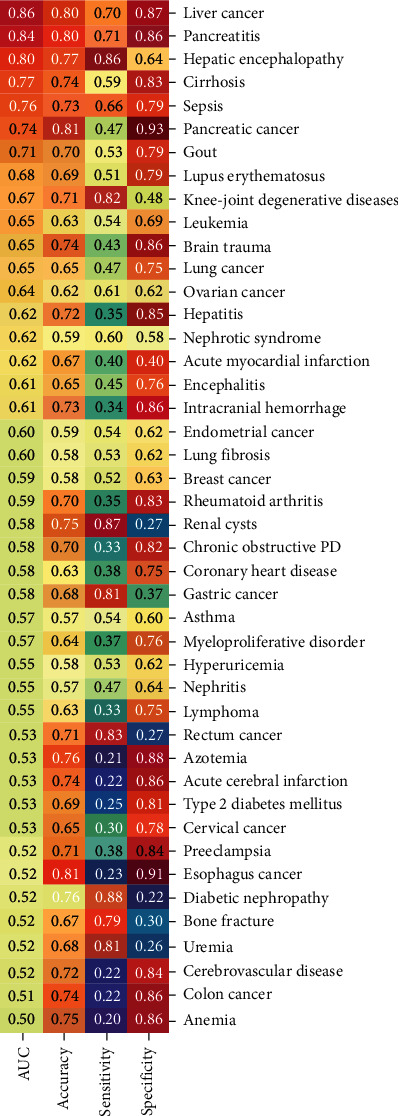
The AUC, accuracy, sensitivity, and specificity of serum GGT activities for 44 types of human diseases. Chronic obstructive PD: chronic obstructive pulmonary disease; AUC: area under the curve.

Lastly, to evaluate the diagnostic properties of serum GGT activities as biomarkers, the receiving operator curve (ROC) analysis was performed for all 44 types of diseases (Figure 4). Based on the ROC analysis, we summarized the area under the curve (AUC); accuracy, sensitivity, and specificity for all diseases are listed in Figure 5 in the descending orders of the AUC values of the diseases.

Among the 44 diseases, 20 had AUCs over 0.60 (ranging from 0.60 to 0.86). The serum GGT activity was the best biomarker for liver cancer with an AUC of 0.86, a sensitivity of 70%, and a specificity of 87%, which were followed by pancreatitis (AUC = 0.84), hepatic encephalopathy (AUC = 0.80), and cirrhosis (AUC = 0.77). In contrast, the serum GGT activities had the lowest AUCs for anemia and colon cancer. The serum GGT activity for pancreatic cancer had the highest specificity (93%) among 44 diseases.

We further observed that all vascular diseases, including acute myocardial infarction, intracranial hemorrhage, encephalitis, acute cerebral infarction, and coronary heart disease, had relatively low sensitivities (0.22-0.45) but high specificities (0.75-0.86) (Figure 5).

## 4. Discussion

GGT has been reported extensively as a widespread disease biomarker [10–26]. GGT is an important risk factor for tumor progression, invasion, and anticarcinogen resistance [18, 25, 26]. Serum GGT levels have been reported as a poor prognostic factor in breast cancer [19], esophagus cancer [20], cervical cancer [21], kidney cancer [22], ovarian cancer [23], stomach cancer [24], nonsmall lung cancer (NSCLC) [10], and colorectal cancer [11]. Increased serum GGT levels have also been reported for heart disease, diabetes, Kawasaki disease, renal insufficiency, hyperuricemia, and inflammation [12–16]. In respiratory diseases, Bozkus et al. demonstrated that serum levels of GGT might contribute to the grading of the severity of chronic obstructive pulmonary disease (COPD) as a biomarker of oxidative stress [17]. There is a strong correlation between high serum levels of GGT and cardiovascular events with COPD [17]. The metabolic syndrome and elevations of ALT and GGT may worsen the atherogenic state [27]. Oxidative stress is when reactive oxygen species (ROS) dominate the systems [28]. The published studies have shown that increased serum GGT are independent biomarkers of the activation of systemic inflammation and increased oxidative stress [10–26].

The data shown in Table 1 and Figures 1–5 suggested that most diseases were associated with increased GGT median values, thus increasing oxidative stress. In contrast, knee-joint degenerative disease, gastric cancer, anemia, renal cyst, cervical cancer, and preeclampsia were associated with decreased GGT median values, suggesting the overall antioxidant stress. Moreover, not only Q25 to Q75 but also Q2.5 and Q97.5 GGT values for all six category diseases significantly exceeded the healthy control in both directions (Figure 2). These observations indicated a swing between oxidative and antioxidative stresses, marked by increased and decreased GGT values, was a common characteristic in disease states.

Our laboratory has published over 20 manuscripts [29, 30] to understand the meaning of different blood test results in various human diseases, including lactate dehydrogenase (LDH), another potential redox biomarker [31]. LDH plays an essential role in glycolysis and gluconeogenesis by catalyzing the reversible conversion of lactate to pyruvate with concomitant interconversion of NADH and NAD^+^ as an oxidoreductase [32]. However, our results indicate that increased LDH in blood circulation results from tissue damage since LDH is a housekeeping protein expressed inside all living cells. Among 48 diseases included in the LDH study, gout had the lowest median LDH levels. Gout is associated with increased uric acid levels. Since uric acid is a potent antioxidant and over half of the antioxidant capacity in human blood circulation comes from uric acid [33], we have compared the uric acid levels among different human diseases. Our results do not support that uric acid is a redox biomarker (the manuscript is in preparation). Our published data showed that most blood tests are indicators of systems malfunction based on the mean, median, and *P* values of the 17 clinical blood test results from 1.4 million clinical samples among 64 human diseases [30, 34, 35]. We found that only GGT qualified as a redox biomarker thus far based on its extracellular location and biological function and facts reported before us [10–26].

Human GGT is encoded by a polygene family composed of 7 different genes, including pseudogenes located on chromosome 22q11 [36]. GGT1 and GGT5 act on gamma-glutamyl residue reversibly from various gamma-glutamyl residue-containing compounds [37]. The cleaved gamma-glutamyl residue is transferred to a variety of substrates [38], which are not limited to glutathione (GSH), GSH disulfide (GSSG), the GSH-xenobiotics made by glutathione-S-transferases, leukotriene C_4_, S-nitroso-GSH [39], drug metabolites, and neuroactive compounds [40–43]. Thus, GGT further expands its enzymatic activity by generating diverse compounds with other physiological effects.

GGT is a glycosylated protein expressed in many tissues, including the bile duct, kidney, pancreas, spleen, gallbladder, brain, seminal vesicle, and heart. GGT present in blood circulation is a high molecular weight of 2000 kDa b-GGT form related to exosomal vesicles [44, 45]. For decades, the increased GGT activity in blood circulation has been regarded as a biomarker for hepatobiliary diseases. It is believed that b-GGT is derived from dead liver cells due to liver injuries [46]. However, b-GGT can be produced by any tissue other than the liver [47].

Located on the cell membrane, GGT catalyzes the degradation of extracellular GSH, allowing cysteine-glycine and cysteine to be available for intracellular synthesis of GSH. Glutathione exists in reduced (GSH) and oxidized (GSSG) states. GSH functions as an essential antioxidant; GGT keeps extra- and intracellular GSH in check. However, possessing both antioxidant and prooxidant activities is not specific for GSH and GTT. For example, the antioxidant vitamin C also has prooxidant activity by generating ROS when Fe(III) is reduced to Fe(II) [48]. Nevertheless, GGT represented a natural and ideal redox status biomarker for different diseases based on the data shown in Figures 4 and 5.

Many clinical trials on antioxidant supplements indicate that either these compounds have no beneficial effects or cause an increase in mortality [49]. Antioxidative stress, the fundamentally opposite of oxidative stress, is due to the overwhelming amount of antioxidants that interfere with the immune system's ability to attack pathogens. Our data in Table 1 and Figures 1–3 clearly showed that oxidative stress and antioxidative stress were associated with all 44 diseases at certain times for certain patients. Thirty-eight out of 44 diseases were dominated by oxidative stress. In contrast, six diseases were overwhelmed by antioxidative stress. Using GGT activity levels to judge the redox status of each patient might be helpful for future personalized patient care.

Oxidative stress is known to contribute to cirrhosis. In oxidative stress, oxidized GSH increases, and hepatic expression of GGT is induced [50]. Thus, hepatic GGT is thought to defend against DNA damages caused by oxidative stress. Unexpectedly, the serum GGT as a biomarker for pancreatic cancer had the highest specificity (0.93, Figure 5) among 44 diseases. Pancreatic cancer had a high degree of malignancy. Early diagnosis and surgical treatment could significantly improve the survival rate of patients with pancreatic cancer. Sensitive and specific biomarkers for pancreatic cancer are needed. We are in the process of testing if GGT could serve as a biomarker for the early detection of pancreatic cancer.

Liver cancer was the fourth leading cause of death among all cancers worldwide [51]. The significant risk factors for liver cancer were liver cirrhosis, alcohol abuse, and hepatitis B and C virus infections [52–54]. Previous studies had shown that GGT levels of liver cancer, hepatitis, and cirrhosis are higher than those in the healthy control [55–57], which was consistent with our results. Remarkably, Figure 3 shows that almost all liver diseases were clustered at the upper right side of the chart, while autoimmune diseases were clustered at the low right side. Moreover, the GGT activity distribution in different diseases had different clustering modes based on the two-component analyses (Figure 4), indicating that variations in circulating GGT activities were regulated differently in different categories of diseases. Thus, understanding the significance of the clustering phenomena would provide new insight into the regulation of redox status in each category of diseases.

The limitations of this study were several folds. First, the study was retrospective, and the numbers of GGT test results for each disease varied greatly, mainly depending on the disease's prevalence. Secondly, the GGT values may change with several variables, such as the particular stages of disease development and therapeutic interventions. We did not make such distinctions in the current study. Instead, we included 95% of data collected for each type of disease for analysis. Such an approach was not conventional but had advantages, which allowed us to rediscover several unknown properties of GGT shown in Table 1 and Figures 1–5. Thirdly, the mechanism associated with increased or decreased GGT activities for specific diseases was still lacking. However, several issues raised from the current findings could be addressed in prospective clinical trials. Further studies that establish oxidative stress and GGT activities may clarify the role of GGT as a redox biomarker for human diseases.

## 5. Conclusion

Most diseases were associated with oxidative stress with increased GGT median values. Moreover, all diseases were associated with over up- and downregulating GGT activities or fluctuated between oxidative and antioxidative stresses during disease progression. Liver- and pancreas-related conditions were outstanding in over-upregulating, while cancers and blood-related diseases were outstanding in downregulating GGT activities in blood circulation. Thus, circulating GGT was a long-sought biomarker of the redox status of different illnesses and would be a valuable tool in monitoring oxidative and antioxidative stresses at the whole-body level to guide the treatment.

## Figures and Tables

**Table 1:** Serum GGT activities (U/L) in 44 different types of clinical defined human diseases and the healthy control.

GGT	# of cases	Mean (SD)	Median (IQR)	*P* value
Healthy controls	132357	21.9 (14.3)	17.0 (12.0, 26.0)	—
Liver cancer	299	123.1 (136.9)	72.0 (33.0, 153.5)	<0.001
Cirrhosis	9656	72.0 (71.3)	46.0 (23.0, 94.0)	<0.001
Hepatitis	7678	41.8 (44.5)	24.0 (14.0, 49.0)	<0.001
Pancreatitis	1846	122.4 (134.1)	69.0 (32.0, 164.8)	<0.001
Pancreatic cancer	1160	139.0 (197.5)	40.0 (18.0, 181.0)	<0.001
Hepatic encephalopathy	91	85.1 (91.0)	38.0 (24.0, 133.0)	<0.001
Brain trauma	661	55.6 (60.0)	32.0 (16.0, 72.0)	<0.001
Intracranial hemorrhage	3861	43.1 (45.7)	25.0 (15.0, 51.0)	<0.001
Encephalitis	560	38.6 (40.8)	23.0 (15.0, 43.3)	<0.001
Acute cerebral infarction	9671	27.6 (22.0)	20.0 (14.0, 32.0)	<0.001
Cerebrovascular disease	4758	24.8 (17.8)	19.0 (14.0, 29.0)	<0.001
Lung fibrosis	336	37.9 (34.5)	25.0 (17.0, 44.5)	<0.001
Lung cancer	10930	30.6 (23.0)	23.0 (16.0, 36.0)	<0.001
Chronic obstructive PD	1651	34.7 (36.3)	22.0 (15.0, 38.0)	<0.001
Acute myocardial infarction	2644	36.8 (31.1)	26.0 (16.8, 45.0)	<0.001
Coronary heart disease	22117	29.9 (23.9)	22.0 (15.0, 35.0)	<0.001
Esophagus cancer	4024	39.2 (41.3)	23.0 (15.0, 46.0)	<0.001
Colon cancer	6644	28.0 (26.5)	19.0 (13.0, 32.0)	<0.001
Rectum cancer	8501	26.4 (25.5)	18.0 (12.0, 29.0)	<0.001
Gastric cancer	13520	27.2 (29.7)	17.0 (11.0, 29.0)	<0.001
Nephrotic syndrome	3852	33.2 (30.3)	23.0 (15.0, 39.0)	<0.001
Azotemia	498	27.7 (22.6)	20.0 (14.0, 32.0)	<0.001
Nephritis	2157	25.5 (21.7)	19.0 (12.0, 29.0)	<0.001
Diabetic nephropathy	605	29.3 (37.3)	18.0 (12.0, 28.0)	<0.001
Uremia	6551	25.0 (20.2)	18.0 (12.0, 29.0)	<0.001
Renal cyst	525	19.5 (12.3)	16.0 (11.0, 23.0)	<0.001
Ovarian cancer	2355	23.8 (16.2)	19.0 (14.0, 28.0)	<0.001
Endometrial cancer	1220	21.4 (13.7)	17.0 (13.0, 24.0)	<0.001
Breast cancer	5508	21.5 (15.1)	17.0 (13.0, 24.0)	<0.001
Cervical cancer	2273	17.9 (11.0)	15.0 (11.0, 21.0)	<0.001
Sepsis	115	54.0 (56.3)	38.0 (22.0, 61.0)	<0.001
Leukemia	5572	35.1 (30.7)	24.0 (15.0, 43.0)	<0.001
Myeloproliferative disorder	1303	32.2 (29.7)	22.0 (15.0, 36.5)	<0.001
Lymphoma	4578	26.1 (19.7)	20.0 (14.0, 31.0)	<0.001
Anemia	2071	23.9 (20.9)	16.0 (12.0, 27.0)	<0.001
Asthma	605	31.4 (32.5)	20.0 (14.0, 33.0)	<0.001
Lupus erythematosus	1339	35.6 (41.8)	20.0 (13.0, 38.5)	<0.001
Rheumatic arthritis	506	31.2 (34.5)	18.0 (12.0, 32.0)	<0.001
Gout	1606	50.1 (39.1)	38.0 (23.0, 62.8)	<0.001
Hyperuricemia	156	33.7 (28.7)	23.0 (14.0, 38.0)	<0.001
Type 2 diabetes mellitus	11629	26.9 (22.6)	19.0 (14.0, 31.0)	<0.001
Bone fracture	1788	24.7 (21.6)	17.0 (12.0, 29.3)	<0.001
Preeclampsia	993	20.6 (20.6)	13.0 (8.0, 25.0)	<0.001
Knee-joint degenerative diseases	445	14.6 (10.3)	12.0 (8.0, 18.0)	<0.001

Healthy control is bolded to make an easy comparison. SD: standard deviation; IQR: interquartile range (Q25 and Q75); Chronic obstructive PD: chronic obstructive pulmonary disease; KJDD: knee-joint degenerative diseases.

## Data Availability

All data files are available upon request. Correspondence and requests for the data files should be addressed to Z.L. and L.Z.

## Additional Points

*Data Analysis*. Correspondence and requests for detailed data analysis should be addressed to Z. L., and L.Z.
